# A New Look at the Effects of Engineered ZnO and TiO_2_ Nanoparticles: Evidence from Transcriptomics Studies

**DOI:** 10.3390/nano12081247

**Published:** 2022-04-07

**Authors:** Shuyuan Wang, Harri Alenius, Hani El-Nezami, Piia Karisola

**Affiliations:** 1 School of Biological Sciences, University of Hong Kong, Hong Kong Island, Hong Kong, China; wangsy@connect.hku.hk; 2 Human Microbiome Research (HUMI), Medical Faculty, University of Helsinki, 00014 Helsinki, Finland; harri.alenius@helsinki.fi; 3 Institute of Environmental Medicine (IMM), Karolinska Institutet, Systems Toxicology, 171 77 Stockholm, Sweden; 4 Institute of Public Health and Clinical Nutrition, University of Eastern Finland, 70211 Kuopio, Finland

**Keywords:** transcriptomics, engineered metal nanoparticles, titanium dioxide, zinc oxide, animal models (in vivo), cell cultures (in vitro), (eco)toxicology, zebrafish, *C. elegans*, *Arabidopsis thaliana*

## Abstract

Titanium dioxide (TiO_2_) and zinc oxide (ZnO) nanoparticles (NPs) have attracted a great deal of attention due to their excellent electrical, optical, whitening, UV-adsorbing and bactericidal properties. The extensive production and utilization of these NPs increases their chances of being released into the environment and conferring unintended biological effects upon exposure. With the increasingly prevalent use of the omics technique, new data are burgeoning which provide a global view on the overall changes induced by exposures to NPs. In this review, we provide an account of the biological effects of ZnO and TiO_2_ NPs arising from transcriptomics in in vivo and in vitro studies. In addition to studies on humans and mice, we also describe findings on ecotoxicology-related species, such as *Danio rerio* (zebrafish), *Caenorhabditis elegans* (nematode) or *Arabidopsis thaliana* (thale cress). Based on evidence from transcriptomics studies, we discuss particle-induced biological effects, including cytotoxicity, developmental alterations and immune responses, that are dependent on both material-intrinsic and acquired/transformed properties. This review seeks to provide a holistic insight into the global changes induced by ZnO and TiO_2_ NPs pertinent to human and ecotoxicology.

## 1. Introduction

The rapid development of nanotechnology holds tremendous potential for wide growth in the applications made of novel nanoparticles (NPs) for various purposes in electronics, medicine, coating materials and even in personal care products (including cosmetics), with more coming online every day [1]. It has been estimated that over 1800 engineered nanomaterial-based products are available in the global market [2], generating a total production volume of nanomaterials of around 11 million tons worldwide [3,4]. Titanium dioxide (TiO_2_) NPs have been extensively produced as a whitening, anti-caking and coloring agent in various products such as paints, cosmetics and foodstuffs [5]. Zinc oxide (ZnO) NPs have excellent semiconducting, light-scattering and anti-microbial properties, which make them a suitable component for electrical and optical devices, cosmetic products and food-packaging materials [6]. At the nanoscale, NPs have a much larger surface area, which confers substantially different and usually somehow enhanced surface properties compared to their bulk-sized counterparts [7]. Although the biological and environmental effects of engineered metal-type NPs have been reviewed in the literature, the majority of published articles have discussed or summarized the responses induced by silver (Ag) NPs [8,9,10,11,12]. There are fewer comprehensive reviews on the impacts of the other two commonly used metal NPs, ZnO and TiO_2_ [1,6,13].

Downscaling of bulk materials allows NPs to gain access to biological organisms and have interactions with biomolecules, sometimes even inside the cells. This ability sometimes makes NPs a desirable vehicle for delivering substances at the cellular level, as demonstrated in the field of nanomedicine, but in other contexts, the enhanced penetration might lead to adverse effects on the living cells, as summarized by others [14,15]. However, the widely used single-endpoint measures are limited and too narrow to capture the generalized outcomes elicited by NPs. Conventional toxicology assays are useful for assessing the end-point effects that are evidenced in phenotypic hallmarks or systemic parameters. However, the data generated from this approach are insufficient to unravel the biological changes occurring at the molecular level. 

The systems biology approach enabled by the rapid development of omics technologies provides a more informative strategy that complements end-point changes with multi-level and comprehensive molecular events upon exposures to NPs [16]. Transcriptomics, in particular, captures changes in global gene expression patterns and strives to provide a holistic understanding of transcriptional mechanisms. Microarray, a fluorescence-based technique, first emerged to enable the quantification of the differential abundance of mRNA transcripts with predetermined sequences and predesigned oligomer probes [17]. Later in the 2000s, the development of next-generation sequencing bloomed and is still being rapidly updated today. RNA-sequencing gives discrete digital read counts as a data output, and it shows enhanced performances in sensitivity, sequence resolution and result accuracy [18]. It provides a high-quality measurement of gene regulation without relying on probe design and prior knowledge of genomic sequences [18]. The differentially expressed genes (DEGs) derived from either microarray or RNA-sequencing are interpreted into meaningful and biologically relevant data via computational tools and a knowledge base of gene functions and the associated pathways, such as Gene Ontology (GO), the Kyoto Encyclopedia of Genes and Genomes (KEGG) and Ingenuity Pathway Analysis (IPA) [19]. A pathway analysis allows us to probe into the interactions between differentially regulated genes and to predict the biological pathways enriched by certain gene networking patterns [19]. 

In this review, we discuss the in vivo and in vitro biological effects of ZnO and TiO_2_ NPs in the field of human and ecotoxicology, as evidenced in transcriptomics studies. 

## 2. Synthetic and Biological Identities of ZnO and TiO_2_ NPs

Metal-based NPs, such as Ag, ZnO and TiO_2_, represent the largest proportion of nanotechnology-derived products [2]. They are incorporated in a myriad of industrial, biomedical and personal care wares and devices, including solar cells, paints, cosmetics, clearing sprays, food additives and therapeutic agents [20,21], owing to their outstanding electrical, plasmonic, optical and anti-microbial characteristics. Exposures to ZnO and TiO_2_ NPs, which are the focus of this review, are likely to occur in humans, and the consequences of such exposures need to be addressed carefully. Despite different routes of exposures, the intrinsic properties of NPs, such as size, surface modifications and dissolution, fundamentally determine the adsorption of biomolecules onto NPs’ surfaces, thereby radically altering their acquired biological identities, cellular interactions and subcellular localization [22,23]. 

### 2.1. Material Intrinsic Properties

The tunability of physicochemical characteristics of NPs lies at the heart of the innovative design of nanomaterials. Modified properties confer new or enhanced performances to achieve a wider or more efficient use of particles in different industrial sectors. Size, shape, surface chemistry and dissolution are the most-studied physicochemical properties of NPs, and they have been well demonstrated to exert significant influences on the biological effects induced by NPs in various experimental set-ups. These important intrinsic properties are described here. 

#### 2.1.1. Size

According to the definition given by the International Organization for Standardization (ISO), NPs are classified as particles having at least one dimension falling in the range of 1 to 100 nm [24], which is the definition we adopted for this article. Additionally, how an NP is defined and its upper size limit rely on its specific application and field of use. For example, NPs utilized in pharmaceutical applications were previously defined as structures that varied in size from 10 nm to 1000 nm [25]. Nowadays, NPs as in vivo delivery vehicles in nanomedicine are often referred to as devices of less than 200 nm in size (i.e., the width of microcapillaries) to allow efficient release of the attached or encapsulated therapeutics [26]. Extremely small NPs (<1 nm) are able to penetrate directly across the cell membrane by passive diffusion, while bigger molecules are more readily taken up via endocytosis mediated by specific receptors or caveolae- or clathrin-coated vesicles or via phagocytosis [27]. The huge reduction in size increases the surface area of each particle and hence renders higher reactivity when compared with the bulk-sized equivalent. On the other hand, it has been suggested that the toxicity of NPs is inversely proportional to the particle size. The high aggregation tendency of NPs also influences the actual toxicity perceived by cells or organisms. Generally, smaller particles demonstrate greater cellular internalization and communications with biomolecules [14,15,28]. Moreover, particle size could affect the biopersistence, distribution and elimination of foreign matters from the biological system. In studies on biological effects of NPs, results show that particle size plays a pivotal role in controlling the location of particle deposition, especially along the respiratory tract [29,30,31].

#### 2.1.2. Surface Modifications

At the time of synthesis, NPs may be given distinct exterior properties with regard to surface charge, coatings and functional groups. Modifications of these surface properties permit finetuning of the toxicity and behaviour of particles in biological systems. In addition to engineered alterations, the particle surface tends to be modified by the dynamic process of bio-corona formation, which consequently affects the ultimate toxicity of NPs. Surface charge is one of the most fundamental properties that influences particle aggregation, cellular uptake and other NP–cell interactions. A number of studies have revealed that NPs carrying positive charge, including ZnO, are more likely to penetrate through negatively-charged cell membranes and genetic materials compared to the same particle of a negative or neutral charge, resulting in greater cytotoxic and genotoxic effects [6,32,33,34]. Cationic NPs are also more easily recognized and removed by the immune cells [35]. In addition to charges, the surface of NPs can be enshrouded with a layer of synthetic coating or extra functionalizations, such as a polyethylene glycol (PEG), amine group (-NH3) and carboxyl group (-COOH) [36,37]. These external molecules are able to reduce particle aggregation via the creation of steric and/or electrostatic repulsion between neighboring particles. Furthermore, they minimize protein interactions with the particles [38], thereby reducing production of reactive oxygen species (ROS) and lowering cytotoxicity [6]. On the other hand, coated NPs are less recognizable by the immune cells due to the “stealth effect”, where coating materials mask the identity of NPs [36], which can lead to problems arising from slower clearance and a higher bio-retention time.

#### 2.1.3. Dissolution

A mix of dissolved metallic NPs and associated ions is produced upon particle dissolution, which requires careful scrutiny of its antimicrobial capacity, cellular toxicity and other biological responses. The rate of dissolution is dependent on particles’ intrinsic properties, such as size, surface properties, surface area and crystallinity, and also external factors, including the pH, ionic strength and the concentration of surrounding media and storage conditions. Metal NPs exhibit different degrees of dissolution in various kinds of media. Generally, they do not dissolve readily in aqueous solutions at a neutral pH, such as in pure water or PBS [39,40,41]. Moreover, purely aqueous media do not reflect a realistic condition in which NPs are in contact naturally. Biological or environmental media with a lower pH and presence of proteins have been found to enhance the dissolution of metal NPs compared to inorganic salt solutions. For example, Ag NPs showed an increased release of Ag ions in a cell culture medium (Dulbecco’s modified Eagle medium) with added fetal bovine serum than in water [42], possibly due to the higher ionic strength and interactions of dissolved ions with cysteine and cysteine-containing proteins present in the cell medium [43,44]. While TiO_2_ NPs showed minimal dissolution in artificial gastric juice (pH 1.5–2), ZnO NPs dissolved readily within minutes of immersion [45], which underscores the profound influence of pH changes on particle dissolution during oral exposures to NPs. Bare metal NPs tend to dissolve into ions more readily than capped counterparts. In addition, solubility is often demonstrated to be inversely proportional to the particle size, as evidenced in studies on Ag, CuO, SiO_2_ and TiO_2_ NPs [46,47,48,49,50]. However, size does not seem to significantly affect particle dissolution in the case of ZnO NPs [48,51,52].

### 2.2. Context-Dependent Properties Relevant for Humans

Upon gaining access to our body, NPs are biologically transformed and conferred with a new identity depending on the formation and composition of another exterior layer, named the bio-corona. Both the bio-corona and inherent properties of NPs determine particles’ fates in the biological system, especially in directing if and how they are recognized by immune cells or interact with other types of somatic cells and cellular components.

#### 2.2.1. Port of Entry

Major ZnO and TiO_2_ NPs exposure routes relevant for humans are (1) ingestion, (2) dermal contact and (3) inhalation. Air exposure occurs mainly under occupational settings during particle synthesis, handling and product manufacturing. Consumers may also inadvertently inhale NPs containing vaporized products, such as cleaning or cosmetic sprays. In addition, uses of nanosized ZnO and TiO_2_-incorporated personal care and cosmetic creams lead to particle entry via dermal contact for the general public. Lastly, ingestion contributes to the principal exposure mode of NP-containing food products, food additives (e.g., E171 (TiO_2_)) and food-packaging materials. It is worth noting that stability and aggregation issues are often associated with oral exposure to NPs upon contact with a multitude of biomolecules and food components and drastic changes in pH. Walczak et al. and Peters et al. have demonstrated that nanosized SiO_2_ and Ag aggregated into larger particles in the gastric environment of an in vitro model that mimicked the human digestion system [53,54]. Surprisingly, these particles reversed back to the nano-size range when they entered the intestinal digestion stage, which was attributed to the shifts in pH and electrolyte concentration. More recently, Zhou et al. also reported a similar improved stability of TiO_2_ and ZnO NPs in intestinal fluid under the influence of oil micelles likely present in digested food [55]. These pieces of evidence suggest that the characteristics and bio-reactivity of metal NPs can be altered during their passage long the gastrointestinal tract. Once NPs gain access to our body, they are first combated by the host defense machinery. However, the unique and nanometric characteristics of NPs may undermine the effectiveness of protective action exerted by immune cells, which invariably complicates the ultimate biological effects of NPs.

#### 2.2.2. Bio-Corona

The formation of the bio-corona enshrouding the surface of NPs is a well-recognized natural phenomenon in biological fluids. The bio-corona is thought to be the acquired identity of NPs in biological systems, and it changes continuously over time, during which there is a dynamic exchange of tightly versus loosely adsorbed corona components in the surrounding media. The bio-corona is primarily composed of proteins, while lipids and sugars may also be present to a lower extent. Albumin, the most abundant type of protein in blood circulation, is the dominating component of the bio-corona. The composition of these coating biological species determines the cellular uptake mechanisms, including adsorbed-opsonin (e.g., albumin and antibodies)-facilitated phagocytosis by immune cells and clathrin/caveolae-dependent endocytosis by other non-specialized types of cells, as reviewed previously [23]. On the other hand, the artificial surface functionalization of NPs can significantly suppress the formation of the bio-corona and hence alter the biological responses elicited by NPs. For instance, hydrophilic PEG can sterically shield NPs from the adsorption of opsonizing molecules in the blood and resist recognition by scavenging immune cells [56]. Ultimately, the particle circulation time, distribution and cytotoxicity depend on the presence and composition of the protein corona of NPs. For example, it has been demonstrated that protein-coated ZnO NPs in serum-containing media exhibited a lower cytotoxicity yet more extracellular ion release when compared with the same particles incubated in serum-free media [57]. In addition, Bianchi et al. observed that lipopolysaccharides, a type of non-protein molecule widely present in the environment and body, adsorbed to TiO_2_ NPs and markedly enhanced the pro-inflammatory signaling pathway in murine macrophages (Raw 264.7 cell line) [58].

#### 2.2.3. Cellular Interactions and Trojan Horse Effect

NPs readily interact with cells and cellular components. Firstly, they are able to penetrate through the cell membrane and impede membrane trafficking activities [59]. Alternatively, any dissolved metal ions could bind to membrane proteins or lipids, increase membrane permeability and enhance the intracellular oxidative stress [60]. When NPs successfully enter the cells, the resulting cytotoxic effect can be ascribed to intracellular metal ion release, which has been suggested to be the most pivotal factor accounting for the toxic potential of 19 kinds of metallic NPs, including ZnO and TiO_2_ [61]. A phenomenon coined as the Trojan horse effect has been proposed as the mechanism underpinning the facilitated metal dissolution in an acidic lysosomal compartment [62], which potentially leads to the malfunction of intracellular proteins and enzymes via ion direct binding, enhanced build-up of oxidative stress, damage of genetic materials and mitochondrial dysfunction [59,63].

#### 2.2.4. Subcellular Localization

Internalized NPs are transported to different subcellular compartments and later digested by lysosomes or removed from the cell via conventional secreting vesicles or unspecific mechanisms [64]. The intracellular trafficking routes usually start with delivery to early endosomes, where some NPs are then transported to recycling endosomes and exocytosed, and others move inwards and fuse with the late endosome and lysosome for biodegradation by enzymes such as lysosomal hydrolases [65]. A portion of NPs may escape from lysosomal digestion to the cytoplasm and accumulate there. Alternatively, the undigested NP may enter the nucleus, mitochondria, endoplasmic reticulum (ER) and Golgi apparatus or leave the cells later. On the other hand, it is also possible that escaped NPs can be re-captured by the autophagic pathway and directed to lysosomal degradation again [66].

### 2.3. Context-Dependent Properties Relevant for the Environment

NPs can enter air, soil and water environments via various routes during manufacturing, transportation, usage or disposal stages [67]. In the environment, NPs undergo physical, chemical and biological transformations, which contribute to their altered physicochemical properties, yielding significantly different effects than the original materials [68,69]. For example, ZnO NPs can be chemically transformed to Zn_3_(PO_4_)_2_ in sludge and biosolids [70]. Compared to pristine nanosized ZnO, the transformed particles exhibit a relatively higher genetic toxicity to mammalian cells due to the greater release of free Zn ions during transformation [71]. Changes in the physicochemical characteristics of NPs can largely affect their bioavailability (i.e., the extent of uptake by organisms or cells) and toxicity [72]. Dissolution of metallic NPs, like ZnO, may decrease the persistence of them in the environment. The transformed NPs may inhibit the growth of bacterial strains, reduce seed germination, decrease plant growth and alter mineral nutrition and photosynthesis [73,74]. On the other hand, transformed NPs may exhibit a decreased toxic potential compared to the pristine form. For example, adsorption of natural organic matter was shown to inhibit the antimicrobial activity of Ag NPs [75]. Additionally, the coexistence of different types of NPs in the same environment can impact the behavior of each other, as evidenced in the study showing a promoting role of TiO_2_ NPs in the ion release of Ag NPs under sunlight [76].

After release into the air environment, aerosolized NPs may agglomerate/aggregate and undergo a redox reaction or photolysis [77]. These reactions largely depend on the properties of pristine NPs and air conditions, including the presence of solid particles, ultraviolet (UV) light, oxygen, ozone and other oxidants (e.g., hydroxyl, nitrate radicals and acid gases). In soils, transformations are largely regulated by soil features and components, such as the water content, texture, ionic strength, organic matter, temperature, pH and biodiversity of organisms [78,79]. These factors can directly or indirectly influence the processes of sorption, aggregation, agglomeration and dissolution [80,81]. Similar to air and soil, transformations in the aquatic environment include various physical, chemical and biological processes, such as aggregation/agglomeration, sorption, dissolution, sulfidation and redox reactions. In reverse, NPs are shown to change community composition, diversity or activity and decrease the biomass of microbes, algae and plants in aquatics. In addition, they are able to induce mortality, malformation formations and changes in behaviour of aquatic vertebrates [82,83,84,85].

Previous studies have reported that certain concentrations of metal NPs, including TiO_2_ and ZnO, were detected in runoff from building facades, sludge from wastewater treatment plants, rivers and sediments and soils [86,87]. A recent study demonstrated that functional chemical groups in particulate matter with an aerodynamic diameter of ≤ 2.5 μm (PM2.5) could attach to the surfaces of TiO_2_ and ZnO NPs by adsorption, leading to changes in particle size, surface charge and functionalization [88]. Another example is that natural organic matter could have electrosteric interactions with ZnO NPs, leading to reduced aggregation of particles [89]. On the other hand, TiO_2_ NPs adsorbed with hydroxyl groups in natural waters have been demonstrated to trigger further interactions with other organic components (e.g., humic acid) in the aquatic system and ultimately cause particle aggregation [90]. For instance, the increase of dissolution rate could result in ZnO NPs being more hazardous in acidic soils [91]. ZnO morphology could also be altered from uniform nanosized spherical particles to anomalous porous particles of a much larger size in the presence of a phosphate solution [92].

## 3. Transcriptomic Profiling Relevant to Human Toxicology

**Figure 1:**
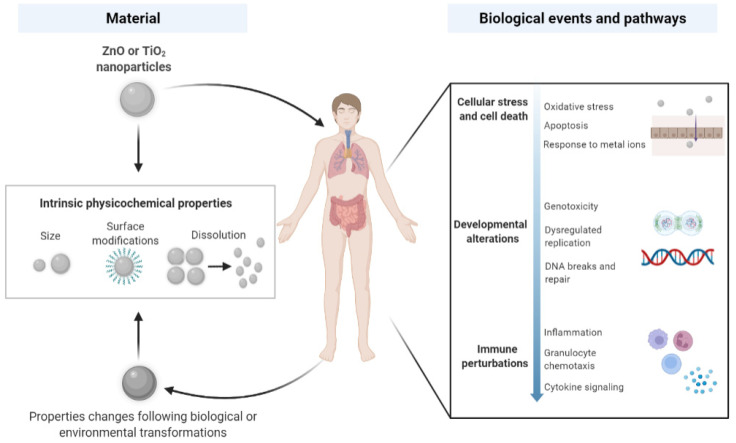
Interactions of ZnO and TiO_2_ nanoparticles with biological systems. Upon human-relevant exposures via ingestion, dermal contact and inhalation, ZnO and TiO_2_ NPs with acquired and/or transformed physicochemical identities, together with material-intrinsic properties, are able to induce various biological processes and pathways. Adapted from “Nanoparticle Interactions with Biological Systems and Vice Versa”, by BioRender.com (2022). Retrieved from https://app.biorender.com/biorender-templates, accessed 25 March 2022.

ZnO and TiO_2_ NPs may cause direct effects to somatic cells and cellular components once they successfully evade from the clearance of immune cells. Beyond the cellular level, they can induce myriads of biological activities in the major exposed organs, gastrointestinal (GI) tract, lungs and skin. Emerging evidence shows that oxidative stress is a primary response to exposures to ZnO and TiO_2_ NPs and/or their ions released [93,94,95], which can further result in genotoxicity due to DNA breaks [96,97,98,99] or apoptotic cell death [100,101,102]. Such cellular stress can also cause perturbations in the immune system and induce inflammation in various tissues [103,104,105,106]. Transcriptomic data have not only corroborated previous findings in conventional studies but also provide new insight into the modulating abilities of ZnO and TiO_2_ NPs in the context of cell and organ homeostasis. Our overview of the current findings regarding the nanosized ZnO- and TiO_2_-induced biological processes and pathways is depicted in Figure 1.

A literature search was conducted on PubMed and Google Scholar using the keywords (and their combinations) “nanoparticles”, “nanomaterials”, “zinc oxide”, “titanium dioxide”, “transcriptomic”, “RNA-sequencing”, “microarray”, “whole genome expression analysis”, “animal”, “cell”, “ecotoxicology”, “in vitro” and “in vivo”. Only studies containing transcriptomic data were assessed for their inclusion in the tables. For the overview of in vitro and in vivo results, the transcriptomic method, model used, material properties, exposure conditions and main transcriptomic findings are summarized in Table 1 and Table 2 for nano-ZnO and nano-TiO_2_, respectively. We marked the main findings of publications, either from the text in results/conclusions or from the DEGs/pathway data tables provided in the original publications.

### 3.1. ZnO and TiO_2_ NPs Exposure In Vitro

Depending on the physicochemical properties of ZnO and TiO_2_ NPs, they are able to evoke cytotoxicity, genotoxicity and immunotoxicity in many in vitro setups. They demonstrate the potential of directly or indirectly interacting with the cell membrane, mitochondria, lysosome and other organelles, leading to a disruption of cellular homeostasis and production of ROS. Despite many shared biological events, TiO_2_ NPs tend to be less bioactive or toxic than ZnO NPs, as shown in some studies [45,132,133].

#### 3.1.1. Cellular Stress and Cell Death

The spontaneous production of ROS by metal-based NPs under UV or visible light has been reported to trigger cell death and inflammation [59]. Once inside the cells, especially positively charged NPs readily interact with organelles such as mitochondria and lysosomes, leading to excessive ROS generation [134]. The oxidative response to ZnO NPs suppressed DNA repair processes but activated the Nrf2 pathway at an early time-point [94], which plays a beneficial role in protecting cells mainly against oxidative damage and cellular dysfunction [135]. Dissolved metal ions also show a high tendency to bind with free radical-scavenging enzymes such as glutathione and superoxide dismutase, which further insults the cellular anti-oxidant capability [134]. These dynamic and counter-balancing events ultimately may direct cells to inflammation, mitochondrial dysfunction and even to apoptotic cell death [136].

ZnO NPs have been shown to have several effects on immune responses, and especially, various metal-ion-related effects are common. In transcriptomic (GO, KEGG or IPA) pathway analyses, a significant enrichment of the terms ‘Response to metal ions’ and ‘Metallothioneins bind metals’ or ‘Translation’, ‘Nonsense-Mediated Decay’, ‘Apoptosis’ and ‘Immune System’ were reported after 6 or 24 h-exposure to ZnO NPs [94,100,107,108], while much less data are available on TiO_2_ NPs. In our own studies, TiO_2_ NPs did not enhance oxidative stress or inflammatory responses in THP-1 monocyte-differentiated macrophages [133]. Metallothioneins are involved in regulating intracellular metal ion concentration and homeostasis, and two different metallothioneins, Mt1a and Mt2A, were among the most upregulated genes after exposure to ZnO NPs [107]. We have noted the importance of metallothioneins also in our own transcriptomic studies in differentiated THP-1 cells [133]. During the longer exposure (24 h) to Zn^2+^ ions, transcripts of the tricarboxylic acid TCA (called also citric acid) cycle were shown to be significantly reduced in human lung epithelial carcinoma cells (A549), indicating major disruption of cellular respiration pathways [94]. Furthermore, with higher ZnO doses, gene expression involved in inflammation, transcription of heat-shock proteins, apoptosis and mitochondrial suffering were increased in PMA-differentiated THP-1 macrophages [100].

The mitochondria are the organelle responsible for energy production within the cells. At the same time, they also have a central role in apoptotic cell death. Recent data demonstrate that besides eliciting caspase activation, mitochondrial outer membrane permeabilization (MOMP) engages in various pro-inflammatory signaling functions [137]. Doumandji et al. found that sirtuin signaling, disturbed protein synthesis with the eIF2 signaling pathway, expression of the membrane damage sensor and stress response with mitochondrial dysfunction were the most affected functions in rat alveolar macrophages (NR8383) after exposure to ZnO NPs [107]. Transcriptomic results from Alsagaby et al. showed similar results, indicating that exposure to ZnO in human chronic myeloid leukemia cells (K562 cell line) changed the anti-oxidant defense system, induced especially mitochondrial-dependent apoptosis (instead of necrosis) and downregulated NF-κB pathway activities. ZnO NPs caused mitochondrial-dependent intrinsic apoptosis in K562 cells, which was probably triggered by oxidative stress-induced DNA damage [108]. Both directly and indirectly Zn-induced dysregulation of the mitochondrion seems to cause cell death and growth [109,110].

#### 3.1.2. Developmental and Hereditary Modifications

Genotoxicity is associated with exposure to metal-based NPs in cells or tissues. Possible mechanisms include NP/NP-released ions binding to DNA directly, NP-induced intracellular ROS or NPs’ interactions with other nuclear proteins that are essential for DNA replication and repair [138]. As a result, DNA molecules are susceptible to dysregulated replication, deformation and chromosomal breaks [138]. In vitro studies have demonstrated that smaller NPs (10 nm) may enter the nuclei directly, whereas disintegration of the nuclear membranes during mitosis assists with the entry of bigger NPs (15-60 nm) [139,140]. Studies have reported the presence of ZnO and TiO_2_ NPs in the nuclei via transmission electron microscopy analysis [96,141].

Depletion of the cellular antioxidant capacity has been shown to contribute to the genotoxicity of ZnO NPs [99]. The direct transcriptomic evidence on the genotoxicity of nanosized ZnO is sparse at the transcriptomic level. Dekkers et al. have shown that exposure to ZnO NPs yields an increase in mRNAs with premature stop codons, which could reflect the increased rate of DNA damage [94]. Furthermore, the expression of DNA repair genes was reduced, including those of the base excision repair, mismatch repair, nucleotide excision repair and double-strand break repair pathways, particularly with Zn ions, micro-sized Zn and nanosized ZnO. The authors concluded that these changes illustrate a progression from adaptive changes, such as metallothionein induction, to the depletion of antioxidants (e.g., glutathione), inhibition of DNA repair and induction of apoptosis [94].

Similarly, owing to oxidative stress, TiO_2_ NPs were shown to trigger DNA breaks and micronucleus formation in skin and liver cells [96,102]. TiO_2_ (E171), which may contain at most 50% of particles in the nano range, previously authorized as a food additive, was no longer considered safe to consume by EFSA in 2016 [142]. In October 2021, the EU decided to ban the use of TiO_2_ as a food additive, starting from early 2022 [143]. After oral ingestion, the absorption of TiO_2_ particles is low, but they can accumulate in the body, and the genotoxicity concerns cannot be excluded. Previous studies show that E171, encompassing NPs and micro-sized particles, induces oxidative stress responses, DNA damage and micronuclei formation in vitro, and recently, a microarray analysis of Caco-2 cell indicated that E171 induced gene expression changes related to signaling, inflammation, the immune system, transport and cancer [115].

#### 3.1.3. Changes in Immune Responses

Due to their size, NPs themselves are usually considered as non-immunogenic, meaning they are not able to trigger immune responses. However, depending on NP surface properties and composition, they may induce versatile immune responses. The particles can associate, bind and form aggregates with available molecules, which together may modulate local or systemic immune responses (being immunosuppressive or immunostimulatory) under healthy and diseased conditions [144].

*Antigen recognition and uptake:* After overcoming the physical epithelial barrier, tissue innate immune cells (such as macrophages, dendritic cells (DCs) and neutrophils) take part in the recognition and uptake of the incoming NPs, mediated by pattern recognition receptors (PRRs), including toll-like receptors (TLRs), and NOD-like receptors (NLRs) on the cell surface of these phagocytes. TLRs are responsible for detecting pathogen-associated molecular patterns (PAMPs), while NLRs are for danger-associated molecular patterns (DAMPs). Nanomaterial-associated molecular patterns (NAMPs), a new type of molecular pattern, also emerge as one of the possible initiators for phagocytosis of novel nanomaterials by the host immunity, in addition to TLRs, opsonic receptors and mannose receptors [145]. The exact recognition mechanism of each type of NP depends on both material-intrinsic properties and the acquired biological identity in different fluids [146,147]. For instance, the components of the bio-corona may act as strong opsonins that aid in efficient phagocytosis via opsonic receptors [144]. Within phagocytes, NPs are intracellularly trafficked to the lysosomal compartment with a low pH, leading to biological degradation via a superoxide/peroxynitrite-driven oxidative pathway or by enzymes such as myeloperoxidase (MPO) and peroxidase [148,149,150].

*Innate immune responses:* Generally speaking, NPs may produce several deleterious consequences while interacting with the innate immunity: the suppression of phagocytosis, induction of cytokine production and activation of the inflammasome complex. NPs may suppress the engulfment of apoptotic cells and microorganisms into the macrophage, leading to a slower or even failed digestion or removal of pathogens [151,152]. Additionally, NPs can directly activate the production of cytokines (e.g., IL-1β, IL-6 and TNF-α) and cause the subsequent inflammatory responses, as seen in the effects produced by TiO_2_ or ZnO NPs in human bronchial epithelial cells and murine astrocytes [101,153,154]. NLRP3 inflammasome complexes are activated if NP-induced lysosomal damage and rupture occurs, accompanied by a release of inflammatory cytokine IL-1β [155,156,157]. Cross-talks between NPs and innate effector cells (e.g., macrophages and neutrophils) contribute to ROS production through the activation of the nicotinamide adenine dinucleotide phosphate (NADPH) oxidase system in inflammatory cells [158], which has been identified as a mechanism underpinning carbon nanotube-induced pulmonary inflammation and fibrogenesis responses [159].

The activation of innate immunity by TiO_2_ NPs was suggested in the study by Jayaram et al. They showed that the exposure of A549 human lung epithelial cells to TiO_2_ (20 nm) activated intracellular ROS, specifically superoxide, along with changes in oxidative stress-related genes, which participate in inflammatory responses, cell surface signaling, and extracellular organization [116]. The TiO_2_-associated DEGs also control the cell cycle that was silenced, causing reduced mediator secretion and cell–cell communication. Together, these may lead to an increased cellular resistance to oxidative metabolism, electron transport and the respiratory chain complex, and metabolic process gene function is enriched. Their experiments suggest that TiO_2_ NPs adapt to oxidative stress through transcriptional changes over multiple generations of cells [116].

*Adaptive immune responses:* In some cases, NP interactions may trigger specific, adaptive responses. Antigen-presenting cells (APCs) like DCs process and present NPs to B or T lymphocytes. For example, TiO_2_ NPs have been shown to enhance the maturation and expression of costimulatory molecules on dendritic cellsl leading to increased proliferation of CD4+ T cells [160]. It has been hypothesized that metal NPs may function as haptens when they are able to conjugate with protein carriers to form larger immunogenic complexes that trigger the elicitation of immune responses and production of antibodies (mainly IgG) [161,162]. Furthermore, previously hidden, conformational epitopes might be revealed, which induce immune responses to the corona-forming proteins [144]. On the contrary, NPs exposure may lead to suppression of T cell proliferation after impeding the differentiation of monocytes into DCs or disturbing DC normal functions [163,164].

Based on the in vitro studies, it is reported that TiO_2_ NPs might compromise the integrity of the blood–brain barrier and cause neuroinflammation. Exposure to noncytotoxic doses (5 μg/mL) of TiO_2_ or Ag NPs had no effects on the transcriptome of T98G human glioblastoma cells [117]. Conversely, the transcriptome of the cells exposed to 20 μg/mL of TiO_2_ NPs revealed autophagy and alterations in several biological processes and molecular pathways, such as “granulocyte chemotaxis”, “response to cytokine”, “inflammation mediated by the chemokine and cytokine signaling pathway”, “B-cell activation” and “T-cell activation” [117]. The results were confirmed by measuring the increased IL-8 production from T98G cultures. In reverse, ZnO NPs did not cause major changes in this study [117].

### 3.2. ZnO and TiO_2_ NPs Exposure In Vivo

ZnO and TiO_2_ NPs may induce local changes in the function of specific organs. Furthermore, due to their ultrasmall size, they are capable of transcending the organ barriers and travelling to non-exposed organs after cellular transcytosis and systemic circulation [165]. While there are already excellent reviews on findings collected from conventional toxicity studies [1,6], the following sections focus on delineating transcriptomic-led studies that have been conducted to reveal the local and/or systemic effects of ZnO and TiO_2_ NPs via the three most-possible exposure routes: ingestion, dermal contact and inhalation.

#### 3.2.1. Ingestion

ZnO NPs exhibit potent antimicrobial property, which renders them widely used in food-packing materials. They can shield food substances from oxygen and moisture in order to maintain their organoleptic qualities [5]. TiO_2_ NPs demonstrate strong light-scattering and whitening effects and have been incorporated into food additives to enhance the color of pastries, confectionery sweets, chewing gum, the coating of chocolates and coffee creamer. It has been reported that nanosized TiO_2_ constitute up to 36% of the food additive E171 [166]. Oral exposures to ZnO and TiO_2_ NPs currently still represent one of the most prevalent human-related exposure routes. Proquin et al. reported that mice that ingested 5 mg/kg E171 (food additive form of TiO_2_ NPs) daily showed a significant downregulation of genes involved in the innate and adaptive immune system, observed as early as on day 7 [118]. Such an immune-inhibitory effect persisted up to day 21, implying a sustained impairment of intestinal immunity. The TiO_2_-induced oxidative stress response was also evidenced in the colonic transcriptome, mediated by the activation of MAPK genes [118]. Furthermore, it is worth noting that the mucin-associated pathway (e.g., O-linked glycosylation) was highly upregulated in the colon [118], suggesting a stimulatory role of TiO_2_ NPs in mucus secretion. 

At the same time, a number of studies have investigated the extraintestinal effects of chronic exposure to ingested ZnO or TiO_2_ NPs. Hu et al. observed a TiO_2_-induced significant elevation of the plasma glucose concentration. The ingestion of TiO_2_ NPs (21 nm, 80% anatase, 20% rutile) for 26 weeks led to enrichment of the same set of genes and pathways, accompanied by the same increase in blood glucose [119]. Although it is well-recognized that ROS production is a possible mechanism contributing to the biological toxicity triggered by NPs, it is less conclusive as to what induces such an increase in the oxidant level. Based on the findings demonstrating a shared ER stress-inducing ability of ZnO and TiO_2_ NPs, Hu et al. suggested it as a mechanism for the observed ROS excess and disruption of blood glucose homeostasis. The liver, along with the kidney and spleen, is a major organ for systemic distribution of orally absorbed NPs [167], supported by its physiological function of detoxification and removal of xenobiotics. It was shown that a significant differential regulation of genes in the liver occurred after 8 or 12 weeks of oral exposure to ZnO NPs at a dose of 25 mg/kg [111], characterized by a noticeable enrichment of genes related to the membrane, endoplasmic reticulum stress, inflammatory responses and generation of ROS. Additionally, a 90-day subchronic oral toxicity study performed by Cui et al. showed that 10 mg/kg of TiO_2_ NPs altered hepatic gene expression associated with inflammatory responses, apoptosis, oxidative stress, cell cycle and differentiation [120]. On the other hand, a 14-day oral administration of ZnO NPs did not induce transcriptional changes related to immunity or cell cycle regulation in the rat liver [112], probably due to a shorter exposure period and the use of different particle-dispersing vehicles (e.g., 5% glucose vs PBS). Additionally, the extraintestinal effects induced by TiO_2_ NPs were studied in the rodent ovary and spleen. The upregulation of genes relevant to oxidative stress, inflammation, ion transport and cell division regulation was common in both organs upon 90-day oral exposures at a dose of 10 mg/kg per day [121,122]. In the mouse ovary, the expression of 10 genes participating in hormonal production and regulation was also increased.

It has been shown that some cells lines well represent responses seen in vivo. Transcriptomic studies in the Caco-2 cell line revealed that E171 induced gene expression changes related to signaling, inflammation, the immune system, transport and cancer [115]. Pure TiO_2_ NPs seem to affect pathways involved in the metabolism of amino acids, creatine and prostaglandin; the urea cycle; the neuronal system; the transport of small molecules (amino acid) and oxidative stress [115]. Two biological processes, the transport of molecules and neuronal system, were shared by E171 and TiO_2_ NPs [115], suggesting that TiO_2_ NPs might have a route to bypass the blood–brain barrier and maybe accumulate into brain tissue.

#### 3.2.2. Dermal Contact

Skin forms the largest barrier for our body against foreign matters and pathogens. One of the environmental stresses that we encounter almost every day is UV radiation. Sunscreen lotions, in addition to physical UV-blocking measures, are able to provide efficient protection against the detrimental effects induced by UV, such as a photosensitive skin rash and even skin cancer. Due to their intrinsic physical properties, ZnO- and TiO_2_-containing sunscreen products are among the most popular type of physical UV filters that directly scatter light and convert UV radiation to harmless infrared light and heat [168,169]. ZnO particles can provide a broader shield against both UVA and UVB waves than TiO_2_ [170]. Nanosized ZnO and TiO_2_, compared to their bulk-sized counterparts, confer the sunscreen cream a transparent and light-weight appearance. On the other hand, these ultrasmall particles have aroused great attention due to their penetration ability across the skin epidermis. Many investigations regarding their local and distant biological effects are still underway.

ZnO NPs are able to accumulate at the hair follicle after topical administration. Ge et al. revealed that 30 nm ZnO NPs caused transcriptional perturbations in cultured mouse hair follicle stem cells and highly enriched genes and pathways related to the regulation of cell communication, apoptosis, cell proliferation/differentiation, RNA synthesis and processing [113]. Beyond skin, the effect of dermal ZnO and TiO_2_ NPs in other organs after possible penetration is still under further investigation. Based on the hepatic transcriptome of mice repeatedly exposed (30 treatments) to ZnO and TiO_2_ NPs on skin, there were no or very few DEGs found in their livers, despite a low-level presence of elemental titanium, probably due to chronic ingestion during daily grooming and licking [114].

We have studied the effects of ZnO exposure during the sensitization or during the challenge of pre-sensitized contact in an allergic individual in a CHS mouse model (data to be published). The skin-swelling effect of the CHS response was markedly inhibited upon topical administration of ZnO NPs during the challenge phase. We saw a significant reduction of local inflammatory cells infiltration and a full abrogation of global innate and adaptive immune responses in the skin transcriptome, suggesting a beneficial effect evoked by ZnO NPs as strong immunosuppressive substances.

#### 3.2.3. Inhalation

The production volume of ZnO and TiO_2_ NPs increases exponentially, reaching thousands of tons of nano-enabled goods produced per year [171]. The inhalation of these NPs during particle synthesis and product manufacturing presents exposure risks to workers, administrative officers and cleaners in the nanotechnology field. Particularly, the circulation of air-borne or surface-settled NPs are promoted during daily clean-up and when staff motility increases [172]. Consumers, such as users of cleaning or sunscreen sprays, may also encounter pulmonary exposure to NP-carrying aerosol, when particles come close to the breathing zone. A number of conventional toxicity testing studies have been carried out regarding the toxic potential of inhaled NPs in lungs. Complementary evidence drawn from transcriptomics-oriented studies further unraveled the biological changes induced by ZnO and TiO_2_ NPs in lungs, which are otherwise missing in the traditional end-point studies.

*Effects of ZnO exposure in lungs:* Plenty of studies have shown that ZnO NPs are able to induce increases in inflammatory cells (e.g., macrophages, neutrophils), cytokines, metallothionein expression and oxidative stress markers (e.g., heme oxygenase-1, SOD and MDA) in BAL fluid or lung tissues [173,174,175,176,177,178,179]. To date, however, there has only been one transcriptomics-led inhalation study on ZnO NPs that has been published. Hadrup et al. measured the levels of DNA strand breaks in BAL fluid and lung and liver tissues by a comet assay [105]. In BAL fluid, increased levels of DNA strand breaks were observed only for coated ZnO at a low-dose and long time after the exposure. In lung tissue, DNA strand breaks were observed for both uncoated and coated ZnO NPs at day 28, whereas no increased levels of DNA strand breaks were observed in liver tissue. These DNA changes in lungs were accompanied with gene expression changes related to unfolded protein responses and the cell cycle G2 to M phase transition during DNA damage checkpoint regulation [105]. In our lab, we performed transcriptomic profiling of a single pulmonary exposure to ZnO NPs via oropharyngeal aspiration in mice, and we followed the lung transcriptome at days 1, 7, and 28 post-exposure (data to be published). We found that ZnO NPs significantly induced the highest number of DEGs on day 1 in lungs, leading to canonical pathways of hypercytokinemia, granulocyte diapedesis, cell-cycle control and interferon signaling. In addition, an unfolded protein response and NRF2-mediated oxidative stress were also the major events induced by inhaled ZnO NPs.

*Effects of TiO_2_ exposure in lungs:* Inflammatory responses in the lungs characterized by the induction of chemotaxis, cytokine signaling and complement cascade pathways have been commonly found in a series of inhalation studies on TiO_2_ NPs, regardless of the chosen route of administration (whole-body inhalation, nasal instillation or intratracheal instillation) or duration of exposure [123,124,125,126]. In addition, it is worth noting that TiO_2_ NPs were able to induce changes in smooth muscle function in lungs. Husain et al. provided transcriptomic evidence pointing out that even a very low dose (18 μg per mouse, corresponding to 1.5 working days based on Danish occupational exposure level) of TiO_2_ NPs, although it did not induce infiltration of inflammatory cells in BAL fluid, was retained in the lungs and significantly downregulated genes related to muscle development/contraction [127] even on 28 days post-exposure. They postulated that the retention of TiO_2_ NPs over time may undermine lung muscle contraction activities via impeding air movement, and as a result, it might lead to the development of lung diseases such as chronic obstructive pulmonary disease and pulmonary fibrosis [127].

Structure- or coating-specific effects have also been studied with inhaled TiO_2_ NPs. Rahman et al. compared the genome-wide alterations induced by different structures (anatase vs rutile) and coatings (hydrophilic vs hydrophobic) of TiO_2_ NPs in mouse lungs [128]. They reported that rutile-structured TiO_2_ was a more potent inducer of transcriptional perturbations than anatase TiO_2_, as supported by much higher numbers of DEGs in the liver, regardless of dosages. Among the rutile, it was shown that a hydrophilic modification imparted greater inflammogenicity compared to the hydrophobic type, as evidenced in their pathway analyses. Nonetheless, both anatase and rutile TiO_2_ induced DEGs associated with inflammatory responses such as cytokine/chemokine signaling, IL-17 signaling, granulocyte adhesion and diapedesis, probably via pathogen pattern recognition mechanisms, including TLR- (for rutile) and NLR-signaling (for anatase). As alluded to earlier, the recognition of NPs can be achieved via TLRs or NLRs, where the former is triggered by bacterial components such as the lipopolysaccharide and peptidoglycan or viral DNA [180], while the latter is initiated by endogenous molecules, including high-mobility group box 1 proteins and heat shock proteins that are released during cellular stress or cell death [181]. Kinaret et al. demonstrated that amination of TiO_2_-NPs led to the strongest inflammation in the airways of mice, while PEGylation substantially inhibited pulmonary toxicity, supported by the transcriptomic profiles of nanomaterials of an identical composition but different coatings [182].

A chronic inflammation-related gene expression pattern was still observed a few weeks after one single exposure to TiO_2_ NPs [123,124,126,128], along with evidence drawn from the histopathological analysis post-exposure. In particular, Chen et al. reported that a single instillation of 0.1, 0.5 or 1 mg of TiO_2_ NPs induced expression of placenta growth factor (PGF) and other chemokines such as CXCL1, CXCL5 and CCL3 in mouse lungs, mimicking an emphysema-like condition (a type of chronic obstructive pulmonary diseases) [124]. On the other hand, Li et al. and Halappanavar et al. sought to recognize the potential effects of inhaled anatase TiO_2_ NPs in lungs after repeated exposure. Treatment with 10 mg/kg of nano-TiO_2_ led to over 500 DEGs after a 90-day consecutive nasal instillation, with the majority being upregulated and involved in immune responses, apoptosis, oxidative stress, metabolic processes, signal transduction and the cell cycle [126]. Additionally, five days after an 11-day repeated whole body inhalation of 42.4 ± 2.9 mg of rutile TiO_2_/m^3^, it led to increases in the expression of gene sets sharing similar functions in the lungs, including acute inflammation, complement cascade, and cytokine/chemokine signaling [125].

*Effects of NPs in distal organs:* In addition to the local effect, efforts have also been put into studying the potential transcriptomic alterations in distal organs. A previous bio-distribution study on inhaled TiO_2_ NPs showed that they were able to translocate to the brain via the nasal cavity [183]. Husain et al. examined TiO_2_ NPs’ influences on the transcriptome profiles of heart and liver tissues 24 h or 28 days after a single intratracheal instillation of TiO_2_. Although a larger amount of TiO_2_ was detected in the liver than the heart, only 63 DEGs were found in the liver, and no specific pathway was enriched, which was in stark contrast to around 500 DEGs present in the heart that significantly perturbed pathways associated with activation of the complement cascade (seven DEGs, including complement factors D and 3), acute phase signaling and inflammatory processes [129]. All transcriptional changes were reversed back to the baseline level when observed at the day 28 post-exposure time-point, suggesting an efficient resolution of TiO_2_-induced inflammatory responses in the heart. It is important to note that Husain et al. also found out that the lectin pathway (microbes-initiated) may be the most possible mechanism explaining the complement activation triggered by TiO_2_ NPs in the cardiovascular system. The complement system encompasses a family of proteins that, when activated, opsonize foreign matters such as NPs, pathogens and damaged cells to direct phagocytosis and recruit more inflammatory cells to the site of activation [184].

Lastly, we found two transcriptomic studies that have been carried out to shed light on the developmental toxicity induced by TiO_2_ NPs in mouse newborns’ livers and hearts, caused during the whole-body inhalation of the same particles in their mother [130,131]. In the study of Jackson et al., female offspring livers showed an altered gene expression related to retinoic acid signaling, while the same gene sets were not responsive to such exposure in male offspring [130]. In the heart of progeny that experienced prenatal maternal exposure to TiO_2_ NPs, the canonical pathways associated with inflammatory signaling and organismal development were the most significantly enriched. In particular, they found increased expression of the lymphotoxin beta receptor gene (a member of tumor necrosis factor receptors), upregulation of IL-8 signaling and downregulation of the inhibitor of nuclear factor kappa-B kinase subunit alpha (IKK-α). Taken together, it was suggested that increased activation of NF-κB and IL-8 pathways may be the mechanism for the TiO_2_-induced inflammation in fetal hearts [131].

## 4. Transcriptomic Studies in Environmental Toxicology

**Figure 2:**
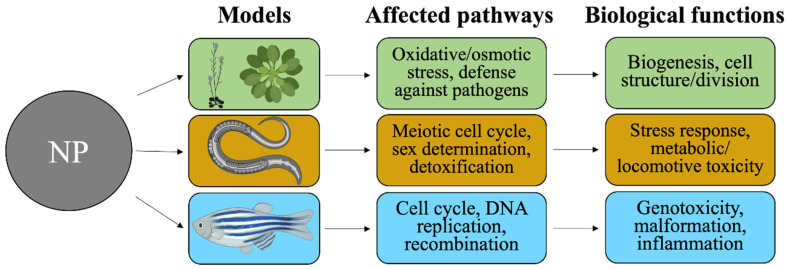
NP-driven environmental effects studied in different ecotoxicology-related models. *Arabidopsis thaliana* plants, *C. elegans* nematodes and *Danio rerio* zebrafish represent soil and aquatic species that are conventionally used for ecological toxicity assessment. Transcriptomic analyses yield DEGs, which identify the major affected pathways and the associated biological functions. Created with BioRender.com (https://app.biorender.com, accessed 25 March 2022).

The extensive production and utilization of engineered ZnO and TiO_2_ NPs increase their chances of being released into the environment and confer unintended biological effects in different environmental organisms upon exposure. It is of high relevance to also understand the most-updated transcriptomic findings on such exposure across representative ecotoxicology species, such as *Danio rerio*, *Caenorhabditis elegans* and *Arabidopsis thaliana*. Our overview of the existing findings (listed in Table 3) regarding the effects of ZnO and TiO_2_ NPs on biological pathways and functions in those representative species is depicted in Figure 2.

The same literature search strategy as mentioned in Section 3 was conducted to screen studies on the effect of ZnO or TiO_2_ NPs on the transcriptome of ecotoxicology-relevant species, with an addition of the keywords “ecotoxicology”, “*Danio rerio*”, “*Caenorhabditis elegans*” and “*Arabidopsis thaliana*”. The eligible studies are listed in Table 3.

### 4.1. Danio rerio

Zebrafish (*Danio rerio*) are commonly used as model animals for testing NP toxicity and biocompatibility, also in high-throughput acute toxicity studies, and for evaluating their value in nanotoxicity assessment [196,197,198,199,200]. Zebrafish are small, transparent, low cost and easy to maintain, with rapid embryogenesis and continuous reproduction [201]. Estimated environmental concentrations of nanosized TiO_2_ and ZnO in aquatic ecosystems range from 0.0007 to 0.0245 mg/mL and 76 µg/L to ≤2 mg/L, respectively [202,203]. Zebrafish can be used to test different NPs and other agents efficiently via multiple routes of exposure, including directly in the water, which is especially relevant for environmental toxicology applications. The combination of a well-established zebrafish model organism, and species-specific oligo microarray platform (Agilent, Santa Clara, CA, USA) and RSEQ strategies provide tools for studies on the molecular mechanisms underlying the adaptive response of fish to NPs, and they aid in the identification of NP-specific genes and signaling pathways in fish.

*ZnO effects in zebrafish adults:* Several studies have reported that ZnO NPs are toxic to zebrafish [201,204,205]. Zhu et al. demonstrated that ZnO NPs induced a concentration-dependent decrease in hatching rates [204]. In addition, previous studies have investigated oxidative stress induced by ZnO NPs in aquatic ecosystems, but their toxicity mechanisms and specific gene biomarkers have remained unknown [206,207,208]. Hou et al. showed that ZnO NPs mainly affected nucleic acid metabolism in the nucleus via alterations in nucleic acid binding [185]. They exposed zebrafish to eight concentrations of ZnO (1-128 mg/L) and found 1434 ZnO-specific DEGs using Agilent zebrafish (V3) gene expression microarrays. KEGG analyses classified the DEGs to the genotoxicity-related pathways “cell cycle”, “Fanconi anemia”, “DNA replication” and “homologous recombination”. Germline inactivation of any of the Fanconi anemia genes causes the disease Fanconi anemia, which leads to bone marrow failure and predisposition to cancer. Hou et al. suggested that based on their pathway results, in addition to double-strand breaks in DNA, NP exposure may also lead to DNA cross-link damage in *D. rerio*. DNA cross-link damage can block the formation of the DNA replication fork and disturb the replication process. The DNA cross-link repair process is relatively complex, and the Fanconi anemia pathway is one effective repair method. Together, these impairments in DNA synthesis and repair can disrupt mitosis or chromosomes by mechanical or chemical binding, leading to enhanced genotoxicity and interference of cell cycle checkpoint functions and the production of mitochondrial ROS.

*ZnO effects in zebrafish embryos and larvae:* Furthermore, pericardial edema and malformations were observed in ZnO NP-exposed embryos [204]. Choi et al. investigated the developmental toxicity of ZnO NPs (0.01, 0.1, 1 and 10 mg/L) to embryonic and larval zebrafish [186]. The principal malformations induced by ZnO NPs were pericardial edema and yolk-sac edema. Gene-expression profiling using microarrays demonstrated 689 DEGs following exposure to ZnO NPs, where six of them were associated with inflammation, and the immune system responded specifically to ZnO NPs. Therefore, ZnO NPs may affect genes related to inflammation and the immune system, resulting in a yolk-sac edema and pericardia edema in the embryonic/larval developmental stages. These results assist in elucidating the mechanisms of toxicity of ZnO NPs during the development of zebrafish. The size-dependent ecotoxicity has also been observed in ZnO NP studies. Kim et al. compared the toxicity of a sublethal concentration of ZnO NPs (LC10, 1 μmol/L) and ZnSO_4_ (LC_30_, 38 μmol/L) in zebrafish after 72 h of exposure [187]. They did not find any DEGs for ZnO NPs after transcriptomics studied in microarray analyses when compared to a non-exposed control group, while ZnSO_4_ yielded hundreds of DEGs. In reverse, according to the identification of lethal doses, ZnO NPs were more toxic than ZnSO_4_ to zebrafish larvae. This discrepancy was explained by the physical properties of the ZnO NPs, which may have been responsible for the increased zebrafish larvae mortality due to the mouth-gaping behavior, which leads to an increased uptake of ZnO NPs [209]. It has been reported that zebrafish mortality was more increased by exposure to ZnO NPs than by bulk ZnO [210], which could indicate that the particle size could be the most important factor controlling ZnO NP toxicity to hatched larvae. When the authors compared the DEGs that were downregulated at the ZnSO_4_ LC10 relative to at the ZnO NP LC10, they found cancer cell differentiation; endocytic transport and genes such as the epidermal growth factor receptor (EGFR), V-Ki-ras2 Kirsten rat sarcoma viral oncogene homolog (KRAS), and phosphoinositide-3-kinase regulatory subunit 6 (PIK3R6) genes were upregulated in ZnO. These data suggest that ZnO NPs induce cell differentiation and pathways associated with the immune system and activate several key genes involved in cancer cell signaling.

*TiO_2_ effects in zebrafish:* TiO_2_ NPs are usually perceived as non-toxic, especially in some short-term studies, and they have already been widely used in many products and applications. The ultimate release of TiO_2_ NPs into the aquatic environment can act as a sink for engineered NPs, while their long-term impact on the environment and human health is still a concern and deserves more research efforts. Jovanović et al. micro-injected TiO_2_ NPs into zebrafish embryos to determine the potential for NPs to change the expression of genes involved in the cross-talk of the nervous and immune systems [191]. After 96 h of follow-up time, 2380 DEGs were induced, which interfered mainly with four areas of organismal and immune functions, including the circadian rhythm, cell signaling through kinase-related activities, exocytosis and trafficking of Golgi vesicles [191]. Altogether, Jovanović et al. concluded that TiO_2_ NPs might cause deregulation of broad physiological and behavioral effects, such as period homolog 2 (Per2) and cryptochromes (1a, 1b and DASH), by the circadian system in aquatic animals [191]. Furthermore, reproductive toxicity has been shown in the chronic exposure of zebrafish to a low dose of nano-TiO_2_ (0.1 mg/L) [192], where a reduced cumulative number of zebrafish eggs was found after 13 weeks of TiO_2_ NPs exposure. The TiO_2_-accumulated fish ovaries suggest a likely penetration of these NPs into the ovary, presumably via blood circulation. In addition, Wang et al. found DEGs that were involved in proteolysis, oxidative stress regulation, metabolism, insulin signaling and apoptosis and oocyte maturation, explaining multi-faceted modes of action for TiO_2_ NPs-mediated disruption of reproduction. In particular, the growth and maturation of stage I follicles were inhibited [192], presumably by altering the expression of several regulators that are critical to this stage of folliculogenesis. At the same time, several up-regulated genes were associated with protein degradation or ROS production, which may reflect the stress responses of ovaries that were dealing with TiO_2_ NPs. All in all, nanosized TiO_2_ in aquatic organisms may lead to alterations in population dynamics and the aquatic ecosystem balance, and thus, it warrants careful scrutiny on toxicity assessment, especially in its long-term impact.

### 4.2. Caenorhabditis elegans

In addition to zebrafish, *Caenorhabditis elegans* can also be used as a representative animal species for ecotoxicological study of the major trophic levels [211,212]. *C. elegans* is a type of nematodes that is abundantly found in the liquid phase of soil or aquatic media, and it feeds on microorganisms in the environment. Furthermore, *C. elegans* has as short life cycle and is easy to grow and handle in the laboratory, which renders it one of the most used in vivo models for toxicological studies, as shown in the toxicity tests of heavy metals in the sediment habitat from a molecular to individual level (e.g., development, reproduction and behaviour) [213,214,215,216]. Acute exposure (within 24 h), prolonged exposure (from the L1 phase of larvae to mature adults; 72 h) or chronic exposure (from adult day 1 to day 10) has been chosen to examine the potential toxicity of specific substances at various concentrations. Acute high-level exposures of ZnO and TiO_2_ NPs in *C. elegans* have been shown to cause detrimental effects at both the lethal and sublethal endpoints, including increased mortality, growth inhibition, impairment of reproduction, decreased locomotion or alterations in gene expression [217,218,219,220,221].

*ZnO effects in C. elegans:* Although prolonged or chronic incubation with ZnO NPs do not significantly affect the mortality, growth or reproduction of *C. elegans* [222,223,224], these particles pose harmful effects with regard to other sublethal markers, including decreased locomotion behaviour (evaluated by body bend and head thrash), increased ROS production and reduced ATP levels [222,223,224]. These previous findings suggest that ZnO and TiO_2_ NPs are associated with oxidative stress and metabolic and locomotive toxicities in *C. elegans*. Reduced ATP levels can be caused by additional energy costs induced by stress responses (detoxification and antioxidant defense mechanisms) or by direct metabolic inhibition by metals or metal ions [225]. Starnes et al. exposed *C. elegans* to a sublethal dose of particulate ZnO NPs or ionic ZnSO_4_ salt for 48 h and revealed that dissolution of ZnO, regardless of forms, may be one of the drivers for the observed shared gene expression pattern, mostly enriched by metal responsive, innate immunity and lysosome pathway-related genes [188]. Nonetheless, there were unique pathways associated with nanosized ZnO only, including amino acid synthesis and metabolism and detoxification processes (ABC transporters). They also demonstrated that although transformed ZnO NPs (phosphatized or sulfurized) drastically reduced the mortality compared to the pristine form, phosphatized ZnO induced the highest number of unique GO terms related to spermatogenesis, meiotic cell cycle and sex determination. These findings suggest that phosphatized ZnO NPs are likely to exhibit distinct toxic mechanisms affecting the reproductive system in *C. elegans* [188].

*TiO_2_ effects in C. elegans:* TiO_2_ NPs have been shown to cause detrimental effects in *C. elegans* larvae and adults. Hu et al. were the first to reveal that TiO_2_ NPs have access to the subcellular compartment of *C. elegans* neurons and consequently negatively affect the axonal growth, as evidenced in phenotypes of shorter axons and inhibited locomotion behavior in a worm thrashing assay [193]. They also explored the cellular mechanisms involved in TiO_2_ NPs toxicity via a microarray. Consistent with the macroscopic observations, various DEGs related to neuronal function were found. Furthermore, although anatase TiO_2_ NPs did not affect larva’s body length as opposed to the rutile type, these particles enriched DEGs that exhibit multiple functions related to metal binding or detoxification, fertility, worm growth and body morphogenesis, such as mtl-2, nhr-257 and clec-70 genes, which are involved in both metal stress responses and worm growth or reproduction [193]. Similarly, the differential regulation of genes related to stress responses, detoxification, metal binding and reproduction have been observed in another microarray study of the effects of TiO_2_ NPs in *C. elegans*, including glutathione-S-transferase, stress resistance regulator and cytochrome P450 [194]. Furthermore, their study showed that anatase TiO_2_ NPs exhibited a greater potential to affect the metabolic pathways than the rutile, while the latter was a stronger inducer of changes in developmental processes [194].

### 4.3. Arabidopsis thaliana

It is generally acknowledged that metallic NP can cause phytotoxicity in various terrestrial plant species, such as wheat, rice and corn [226,227,228]. *Arabidopsis thaliana* is an ideal test plant species for toxicity screening of terrestrially relevant substances, owning to its quick germination process and relatively short life span [229]. Additionally, it exhibits a high sensitivity to potential toxicants, owing to the relatively larger surface area-to-volume ratio of its small seed [230]. Physiological parameters such as decreased biomass, reduced root length, delayed see germination speed, altered nutrient transport and lowered chlorophyll content have been associated with the plant’s response to stress induced by metal NPs (therefore, also identified as phytotoxins) [231,232,233,234,235]. For example, ZnO NPs could impede the chlorophyll synthesis process in wheat leaves and induce size-dependent inhibition of seed germination in *A. thaliana* [226,236]. A couple of modes of action at the biochemical, molecular and morphological levels have been investigated regarding the phytotoxicity of ZnO and TiO_2_, such as ZnO or TiO_2_ NPs-induced genotoxic chromosomal aberrations or DNA breaks [237,238] and lipid peroxidation via ROS production in an onion and *A. thaliana* [239,240]. Although studies by toxicogenomic profiling of *A. thaliana* remain very limited, it was the first plant to have had its genome sequenced in 2000 [241].

*ZnO effects in A. thaliana:* Microarrays were conducted to analyze and compare the gene regulation in *A. thaliana* roots upon a seven-day treatment with ZnO or TiO_2_ NPs [189]. Overall, it was shown that ZnO NPs were able to induce a much greater number of DEGs and associated diverse biological processes, indicating a severe phytotoxic role of ZnO NPs in contrast to mild changes (mainly responses to biotic and abiotic stimuli) in global gene expression triggered by TiO_2_ NPs. Specifically, ZnO NPs significantly reduced the transcription of genes involved in cell organization, biogenesis, translation, nucleosome assembly and microtubule-based process, suggesting the capability of ZnO NPs in altering cell structure, cell division activity and DNA packaging. Additionally, nanosized ZnO exposure downregulated the genes coded for ribosomal proteins mainly involved in electron transport and energy pathways, possibly leading to a reduction in overall protein biosynthesis of the whole plant. On the other hand, adaptive responses to salt, wound, metal ion, oxidative and osmotic stresses, along with defense against pathogens, were strongly upregulated upon ZnO NPs incubation. A later study conducted by the same group elucidated that released Zn ions largely contributed to the toxic effect observed in *A. thaliana* roots based on the similarity in transcriptomic profiles of root samples exposed to ZnO NPs versus ionic ZnSO_4_ [190].

*TiO_2_ effects in A. thaliana:* In addition to studying the roots of mature *A. thaliana*, Tumburu et al. provided a phenotypic and transcriptional understanding of the alterations induced by a 12-day TiO_2_ NPs treatment in the germinants of *A. thaliana* [195]. Interestingly, they showed that TiO_2_ NPs were able to enhance germination, evidenced by an increased percentage of seeds exhibiting hypocotyls and cotyledons and a greater number of germinants with fully grown leaves, which agrees with another study showing promoted germinant growth in wheat after exposure to TiO_2_ NPs [242]. Overall, there were much more upregulated than downregulated DEGs induced by TiO_2_ NPs [195]. Despite the primary upregulation of genes participating in oxidative and osmotic stresses, an array of metabolic processes, including DNA, protein and phytohormone metabolism; tetrapyrrole synthesis in the chloroplast and photosynthesis, were notably upregulated as well, which were believed to synergistically facilitate root growth and development, cell organization and cell differentiation. For example, significant increases in the expression abundance of cell wall proteins (e.g., arabinogalactan-proteins) were noted. The authors suggested that photocatalytic TiO_2_ NPs may promote *A. thaliana* seedling growth and development via elevating the expression of genes that have key roles in photosynthesis and hormonal metabolism [195]. A potential beneficial role of TiO_2_ NPs in the growth of *A. thaliana* remains to be further explored.

## 5. Conclusions

With the increasingly prevalent use of omics techniques, new data are burgeoning to provide a global view on the overall changes induced by exposure to ZnO and TiO_2_ NPs. The variable results reported on particles with different characteristics, concentrations and exposure routes have sometimes yielded even opposite outcomes at different organs. Transcriptomic studies enable us to better track, compare and conclude the shared and particle-specific effects. These findings are essential for the optimization of “safety by design” of NPs for wider uses in human society. It is noticeable that transcriptomics data on diseased in vivo models regarding the gut, skin and lungs are still missing. Research on contrasting the differential effects of ZnO or TiO_2_ NPs in healthy versus immuno-compromised organisms is warranted for evaluating the risk of exposures for vulnerable populations. The transcriptomic approach offers a more detailed view of the cellular and organismal responses after NP exposures in in vitro and in vivo studies. It advances the toxicological understanding and sheds light on the local-oriented and distant effects of original, distributed or transformed NPs in organisms and also in the environment.

## Figures and Tables

**Table 1:** Transcriptomic characterization of exposures to ZnO NPs in in vitro and in vivo studies.

ZnO In Vitro					
Study	Method	Cell Model	Material Properties	Exposure Conditions	Main Transcriptomic Findings (↑ Means Upregulate, Increase, Stimulate; ↓ Means Downregulate, Decrease or Suppress)
[94]	RNAseq	Human lung epithelial carcinoma cells (A549)	Uncoated 42 nm	15 μg/mL for 1, 6 or 24 h	**Enriched terms “response to metal ions”, “metallothioneins bind metals”, “apoptosis” and “immune system” ( at 6 &24 h); ↓ molecules related to DNA repair; Nrf2 pathway was predicted to be activated at 6 h but repressed at 24 h**
[100]	Microarray	Phorbol 12-myristate 13-acetate (PMA)-differentiated THP-1 macrophages	Uncoated, <50 nm	2 or 8 μg/mL for 4 h	**Affected genes involved in metal metabolism, transcription regulation, DNA binding, protein synthesis and structure**; at higher dose, **altered gene expression involved in inflammation, apoptosis and mitochondrial dysfunction**
[107]	Microarray	Rat alveolar macrophages (NR8383)	Uncoatad, 158 nm	4 and 17 μg/mL for 4 h	**Disturbed protein synthesis/homeostasis with the eIF2 and VEGF signaling pathways**, stress response with mitochondrial dysfunction, and sirtuin signaling; **↑ metallothioneins,** genes related to membrane damage sensor, lung fibrosis, and protein synthesis regulator; **↓ stress response mediator**, cell-cycle regulator, and transcription factor
[108]	Microarray	Human chronic myeloid leukemia cells (K562 cell line)	Uncoated, ≤40 nm	40 μg/mL for 15 h	**↑ Genes involved in “response to zinc ions”, “detoxification of inorganic compound”, and “negative regulation of growth”; ↓ genes that regulated “immune responses”, “cell proliferation/migration”, “receptor signaling pathway via JAK-STAT” and “phosphatidylinositol 3-kinase signaling”**; ↑ anti-oxidant defense system, mitochondrial-dependent apoptosis, and ↓ NF-κB pathway
[109]	RNAseq	Human skin cancer cells (A431)	Uncoated, around 500 nm	150 μg/mL for 6 h	**Altered gene expression for pathways in cancer**, alcoholism, environmental information processing including **MAPK, cytokine, TNF signaling pathways**; **↑ genes related to injured or inflamed skin, and ↓ genes of apoptosis/cell cycle/cell survival**
[110]	Microarray	Human monocyte-derived macrophages; Jurkat T cell leukemia derived cell	Uncoated, 15 nm	1 or 10 μg/mL for 6 or 24 h	**Affected cell death, cell growth, immune system development processes**
**ZnO In Vitro**					
**Study**	**Method**	**Animal model**	**Material Properties**	**Exposure Conditions**	**Main Transcriptomic Findings** **(↑ Means Upregulate, Increase, Stimulate; ↓ Means Downregulate, Decrease or Suppress)**
[105]	Microarray (lung)	C57BL/6J BomTac female mice	Uncoated, 100 nm	Intratracheal instillation at 11, 33 or 100 mg/kg once	**Enriched pathways for cell cycle G2 to M phase DNA damage checkpoint regulation, circadian rhythm signaling, protein ubiquitination pathway, unfolded protein response**, and AMPK signaling
[111]	RNAseq (liver)	CD-1 male mice	Around 35 nm	Oral administration at 25 mg/kg for 8 or 12 weeks	**Most significantly enriched Gene Ontology (GO) and Kyoto Encyclopedia of Genes and Genomes (KEGG) pathways involved membranes, endoplasmic reticulum stress and ROS generation**
[112]	RNAseq (liver)	Sprague Dawley female rats	Uncoated, 86.3 nm	Oral administration at 100 mg/kg for 14 consecutive days	**↑ Metabolic process and metal binding in liver**
[113]	RNAseq (cultured skin cell)	CD-1 mice	Around 30 nm	Mouse hair follicle stem cells were exposed at 20 μg/mL for 12 h	**Perturbed genes associated with hair follicle stem cell apoptosis and differentiation**; altered pathways involved in cellular communication and RNA biosynthetic processes
[114]	RNAseq (liver)	Hairless SKH:QS mice	Uncoated, 18.2 ± 0.4 nm	Dermal application at 2 mg/cm^2^ to the head, ears, back, sides and tail, for 30 treatments	No statistically significant DEGs

**Table 2:** Transcriptomic characterization of exposures to TiO_2_ NPs in in vitro and in vivo studies.

TiO_2_ In Vitro					
Study	Method	Cell Model	Material Properties	Exposure Conditions	Main Transcriptomic Findings (↑ Means Upregulate, Increase, Stimulate; ↓ Means Downregulate, Decrease or Suppress)
[115]	Microarray	Undifferentiated Caco-2 cells	E171; Antase, 15–25 nm	1.4 μg/cm^2^ for 2, 4, and 24 h	**E171 and TiO_2_ NPs ↑ genes for inflammation, immune system, transport and cancer; E171 ↑ metabolism of proteins with the insulin processing pathway; TiO_2_ NPs affected pathways involved in metabolism of amino acids, prostaglandin, urea cycle, oxidative stress**; two common biological processes: transport of molecules and neuronal system
[116]	RNAseq	Human lung epithelial carcinoma cells (A549)	Anatase (80%) and rutile (20%), 21 nm	800 μg/mL for 24 h	**↑ Genes related to inflammatory response, cell surface signaling, oxidative stress, extracellular organization**, electron transport, respiratory chain complex, and metabolic processes; **↓ genes that control cell cycle**, secretion and cell–cell communication
[117]	RNAseq	Human glioblastoma cells (T98G)	18 nm	20 μg/mL for 72 h	**Altered biological processes and functions were “granulocyte chemotaxis”, “response to lipopolysaccharide”, “response to cytokine”; enriched pathways “interleukin signaling”, “chemokine and cytokine signaling”, “B-cell activation” and “T-cell activation”**, “cadherin signaling” and “integrin signaling”
**TiO_2_ In Vitro**					
**Study**	**Method**	**Animal model**	**Material Properties**	**Exposure Conditions**	**Main Transcriptomic Findings** **(↑ Means Upregulate, Increase, Stimulate; ↓ Means Downregulate, Decrease or Suppress)**
[118]	RNAseq (colon)	BALB/c male and female mice	E171	Oral administration at 5 mg/kg for 2, 7, 14 or 21 days	**↓ Genes involved in innate and adaptive immune system**; modulated signalling genes involved in colorectal cancer and biotransformation of xenobiotics
[119]	RNAseq (liver)	CD-1 mice	Anatase (80%) and rutile (20%), 21 nm	Oral administration at 50 mg/kg for 26 weeks	**Most significantly enriched GO terms and KEGG pathways included plasma glucose homeostasis, metabolic mechanisms, generation of ROS, endoplasmic reticulum stress, and unfolded protein**
[120]	Microarray (liver)	CD-1 female mice	Anatase, 5–6 nm	Oral administration at 10 mg/kg for 90 days	**Altered gene expression for inflammatory response, apoptosis, oxidative stress, metabolic process, signal transduction, cytoskeleton**, ion transport, cell proliferation, and cell differentiation
[121]	Microarray (spleen)	CD-1 female mice	Anatase, 7 nm	Oral administration at 10 mg/kg for 90 days	**Perturbed gene expression involved in immune responses, apoptosis, stress responses, metabolic processes, signal transduction, cytoskeleton, oxidative stress, ion transport**, cell division, and translation
[122]	Microarray (ovary)	CD-1 female mice	Anatase, 6 nm	Oral administration at 10 mg/kg for 90 days	**Significantly upregulated DEGs involving hormone levels and reproduction, immune and inflammatory responses, transcription, ion transport, regulation of cell proliferation, and oxidoreductase activity**
[114]	RNAseq (liver)	hairless SKH:QS mice	Anatase (80%) and rutile (20%), 21 nm	Dermal application at 2 mg/cm^2^ to the head, ears, back, sides and tail, for 30 treatments	No significant changes
[123]	Microarray (lung)	CD-1 mice	Anatase (80%) and rutile (20%), 21 nm	Intratracheal instillation at 5, 20 or 50 mg/kg once	**↑ Enriched genes related with antigen presentation and induction of chemotaxis of immune cells**; probably caused chronic inflammatory diseases through Th2-mediated pathway
[124]	Microarray (lung)	CD-1 male mice	Rutile, 21 nm	Intratracheal instillation of 0.1 or 0.5 mg once	**↑ Pathways including cell cycle, apoptosis, chemokines, and complement cascades; ↑ genes in placenta growth factor and other chemokines expressions** that may cause pulmonary emphysema and alveolar epithelial cell apoptosis
[125]	Microarray (lung and liver)	C57BL/6BomTac female mice	Rutile, 20 nm, coated with polyalcohols	Whole-body inhalation at 42 mg/m^3^ for 11 days (1 h/day)	**↑ Genes associated with acute phase, inflammation and immune response; associated pathways included cytokine–cytokine receptor interaction**, metabolism, complement and coagulation cascade, hematopoeitic cell lineage, and biosynthesis of steroids
[126]	Microarray (lung)	CD-1 female mice	Anatase, 6 nm	Nasal instillation at 2.5, 5 or 10 mg/kg for 90 days	**↑ Genes involved in immune/inflammatory responses, apoptosis, oxidative stress, cell cycle**, metabolic processes, stress responses, signal transduction, and cell differentiation
[127]	Microarray (lung)	C57BL/6 female mice	Rutile, 21 nm, coated with polyalcohols	Intratracheal instillation at 18, 54 or 162 μg/mouse once	**↑ Inflammatory gene expression; ↓ genes involved in ion homeostasis and muscle regulation**
[128]	Microarray (lung)	C57BL/6J female mice	Anatase, rutile or anatase/rutile; 8, 20 and 300 nm; and hydrophobic or hydrophilic surface modifications	Intratracheal instillation at 18, 54, 162 or 486 μg/mouse once	**Rutile type induced higher number of DEGs relate to inflammataion and acute phase signaling; hydrophilic surface induced higher DEGs; among the anatase, the smallest type showed the maximum response; anatase types enriched inflammatory response, response to wounding,** defense response, chemotaxis; **high dose of anatase TiO_2_ affected cytokine–cytokine receptor interaction, chemokine signalling, NOD-like receptor signalling, p53 signalling**, ataxia telangiectasia mutated signalling, and steroid metabolic process
[129]	Microarray (liver and heart)	C57BL/6 female mice	Rutile, 21 nm, coated with polyalcohols	Intratracheal instillation at 162 μg/mouse once	**↑ Complement cascade and inflammatory processes in heart** for particle opsonisation and clearance; mild changes in gene associated with acute phase responses in liver
[130]	Microarray (liver)	C57BL/6BomTac female mice	Rutile, 21 nm, coated with polyalcohols	Whole-body inhalation at 42 mg/m^3^ for 10 days (1 h/day) during gestation	**Altered gene expression related to the retinoic acid signalling pathway in the female newborn livers; associated pathways related tissue development and vitamin, mineral and lipid metabolism**
[131]	RNAseq (heart)	Sprague Dawley female rats	Anatase (80%) and rutile (20%), 21 nm	Whole-body inhalation at 10 mg/m^3^ for 7–8 days (4–6 h/day) during gestation	**Altered pathways involved in inflammatory signaling and organismal development; ↓ protein kinase B (AKT) signaling; ↑ IL-8 signaling**

**Table 3:** Transcriptomic characterization of exposures to ZnO and TiO_2_ NPs in ecotoxicology-related models.

**ZnO**					
**Study**	**Method**	**Ecotox Model**	**Material Properties**	**Exposure Conditions**	**Main Transcriptomic Findings** **(↑ Means Upregulate, Increase, Stimulate; ↓ Means Downregulate, Decrease or Suppress)**
[185]	Microarray	Zebrafish	<50 nm	4.8 mg/L for 96 h	**Mainly affected nucleic acid metabolism via altering nucleic acid binding; enriched KEGG pathways included “cell cycle”, “DNA replication”, and “homologous recombination”**
[186]	Microarray	Zebrafish	Uncoated, 20–30 nm	0.01, 0.1, 1 or 10 mg/L for 96 h post-fertilization	**↑ Genes for inflammation and the immune system; toxicological pathways included cytokine-cytokine receptor interactions and the intestinal immune network for IgA production**
[187]	Microarray	Zebrafish larva	Uncoated, 10–30 nm	1 or 4 μmol/L for 72 h post-fertilization	**↑ Cell differentiation and pathways associated with the immune system; ↑ several key genes involved in cancer cell signaling**
[188]	Microarray	*Caenorhabditis elegans*	Pristine, phosphatized or sulfidized, 30 nm	0.7 mg/L (ZnO), 7.5 mg/L (pZnO) and 7.5 mg/L (sZnO) for 48 h	**Induced DEGs related to metal responsive genes; enriched pathways for protein biosynthesis (Aminoacyl-tRNA biosynthesis) and associated with detoxification (ABC transporters)** were shared among pristine and one or both transformed ZnO NPs
[189]	Microarray (roots)	*Arabidopsis thaliana*	Uncoated, <100 nm	100 mg/L for 7 days	**Mainly perturbed genes involved in stress responses to abiotic (oxidative, salt, water deprivation) and biotic (wounding and defense to pathogens) stimuli; ↑ genes involved in cellular metal ion homeostasis and transport, and enzymes against oxidative stress; ↓ genes related to cell organization and biogenesis, translation, nucleosome assembly and microtubule-based process**
[190]	Microarray	*Arabidopsis thaliana*	Uncoated, 20 nm	4 mg/L for 7 days	**↑ Genes for stress responses (e.g., to salt, osmotic stress or water deprivation)**, responses to pathogens, oxidative stress, transcription factors, and transporters; **↓ genes involved in cell organization and biogenesis, nucleic acid metabolism**, ribosomal proteins, cell wall modification and cell growth
**TiO_2_**					
**Study**	**Method**	**Ecotox Model**	**Material Properties**	**Exposure Conditions**	**Main Transcriptomic Findings** **(↑ Means Upregulate, Increase, Stimulate; ↓ Means Downregulate, Decrease or Suppress)**
[191]	Microarray	Zebrafish embryos	Anatase, 25 nm	Microinjections of 8.5 ng/g	**Interfered pathways related to circadian rhythm, cell signaling through kinase-related activities, trafficking of Golgi vesicles, immune function**, and exocytosis
[192]	Microarray (ovary)	Zebrafish	Anatase, <25 nm	0.1 and 1 mg/L for 13 weeks	**Perturbed expresssion of genes involved in proteolysis, oxidative stress regulation, metabolism, insulin signaling, apoptosis and oocyte maturation**; ↑ genes associated with protein degradation or ROS production
[193]	Microarray	*Caenorhabditis elegans*	Anatase, 32 nm	200 μg/mL for 72 h	**Affected genes involved in metal binding/detoxification, fertility and reproduction, worm growth, body morphogenesis, and neuronal function**
[194]	Microarray	*Caenorhabditis elegans*	Anatase (83%) and rutile (17%), 34.1 nm; anatase: 5.9–16.2 nm; rutile: 12.6–68.9 nm	0.01, 0.1, 1 and 10 mg/L for 24 h	**Altered regulation of anti-oxidant system, stress resistance regulator and embryonic development; anatase type greatly influenced metabolic pathways whereas rutile particles significantly affected developmental processes**
[189]	Microarray (root)	*Arabidopsis thaliana*	Anatase (80%) and rutile (20%), 21 nm	100 mg/L for 7 days	**Mild changes**, primarily responses to biotic and abiotic stimuli
[195]	Microarray	*Arabidopsis thaliana* germinants	Anatase (80%) and rutile (20%), 21 nm	500 mg/L for 12 days	**↑ Genes related to metabolic processes** (DNA metabolism, hormone metabolism, triterpenoid biosynthesis and photosynthesis, indole glucosinolate metabolism, tryptophan catabolism), **root development and cell differentiation, ion transport, and redox reaction; ↓ genes related to respiratory burst, responses to stress, hypoxia, and immune responses**

## Data Availability

No new data were created in this study. Data sharing is not possible in this article.
